# The Grave Consequences of Late Presentation and Management of Rare Double Left Ventricle Chamber With Cardiomyopathy

**DOI:** 10.7759/cureus.9404

**Published:** 2020-07-26

**Authors:** Mohammed Al-Musawi, Slee Yi, Suhad AlOmaishi, Levonti Ohanisian, David Rubay

**Affiliations:** 1 Surgery, University of Colorado Anschutz Medical Campus, Aurora, USA; 2 Surgery, Charles E. Schmidt College of Medicine, Florida Atlantic University, Boca Raton, USA; 3 Internal Medicine, Life Alliance Organ Recovery Agency, University of Miami, Miami, USA; 4 Orthopaedic Surgery, Morsani College of Medicine, University of South Florida, Tampa, USA

**Keywords:** cardiomyopathy, double-chambered ventricle, congenital anomaly, dclv

## Abstract

A double‑chambered left ventricle is a rare congenital anomaly. We present the case of a 26-year-old man with such anomaly who presented with congestive heart failure. After this diagnosis was confirmed with echocardiography, surgical removal of the anomalous band and replacement of the regurgitant deformed mitral valve were performed. Postoperatively, the patient deteriorated, and no corrective response was associated with surgery. Herein we discuss what we have learned from this rare case and how it may apply to the management of similar cases in the future.

## Introduction

A double‑chambered left ventricle (DCLV) constitutes an extremely rare congenital malformation, and compared with a double right ventricle (RV), is a more common pathology [1-4]. A combination of these two in a patient has also been described, although even rarer [5]. A literature search found that only a few case reports of a DCLV have been described to date in which the left ventricle (LV) anomaly is characterized by the subdivision of the LV into two chambers by an abnormal septum or muscle band [6,7]. The few available reported cases show that cardiomyopathy is often an associated pathology [3,7]. The degree of the clinical symptoms of DCLV is associated with the size of the access hole between the main LV chamber and the accessory chamber and the heart function of the patient [1]. Small access holes or ventricular dysfunction can cause palpitations, arrhythmia, and left heart failure [8]. Treatment is generally not required if the patient lacks the clinical manifestations of LV insufficiency; otherwise, surgical treatment is required if left cardiac insufficiency is present [9].

## Case presentation

**Figure 1:**
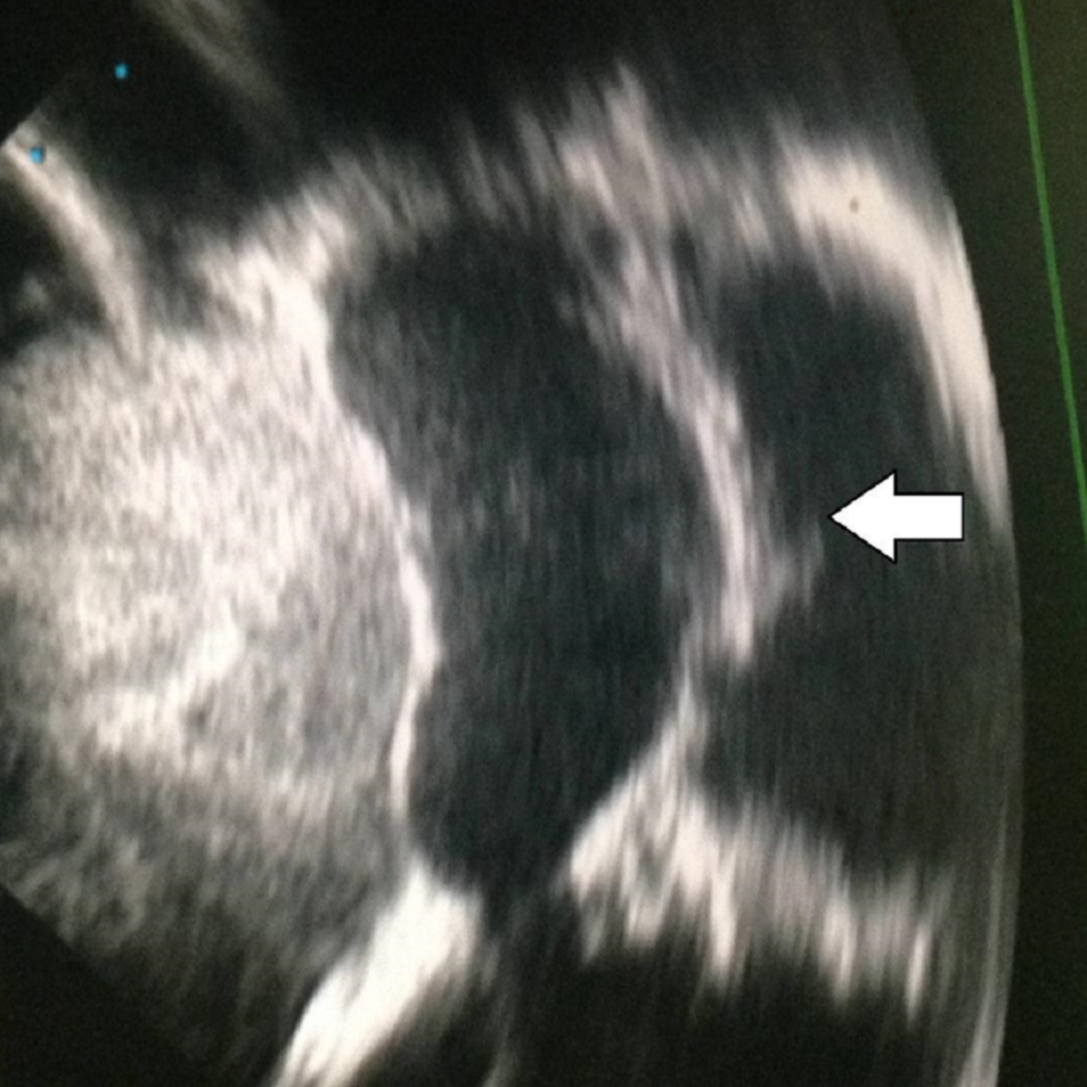
Echocardiographic image showing the transverse band in the left ventricular chamber

Our patient is a 26-year-old man who presented with fatigue and exercise intolerance. An echocardiogram demonstrated a clear band dividing the LV into two chambers (Figure 1) communicating through small orifices in the band, which is tethering to a single papillary muscle of the LV. There was a moderate to severe eccentric regurgitant jet through the mitral valve. The patient had severe heart failure with low ejection fraction (28%).

**Figure 2:**
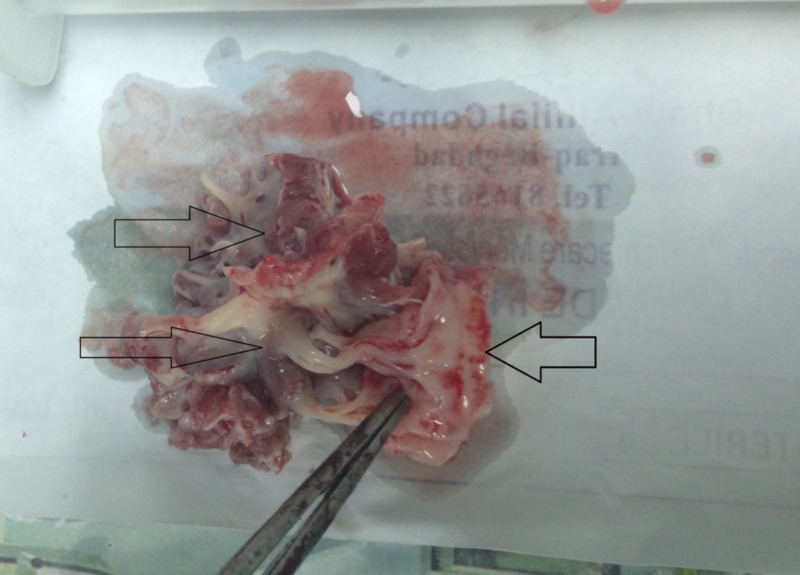
The resected fibromuscular band showing the attachment to the single mitral leaflet

Anti-failure management started with loop diuretics, thiazides, and digoxin for one month, and the patient was prepared for open heart surgery to remove the band and restore the single cavity morphology of the LV. Under general anesthesia, surgery was performed through a median sternotomy approach, aortocaval cannulation, aortic cross-clamp, and cardioplegic arrest. A conventional left arteriotomy incision and entering the LV via the mitral valve was performed. A clear transverse fibromuscular band was seen tethered to the anomalous papillary muscle, which lacked the usual anatomy of separate anterior and posterior components. Using fine scissors, we excised the fibromuscular band and separated it from the papillary muscle (Figure 2). It was inevitable to cut the anomalous single papillary muscle, which was matted to the anomalous fibromuscular ridge. 

Single chamber LV was restored, and the mitral valve was replaced with a mechanical one. The heart did not beat spontaneously after removing the aortic cross-clamp. Therefore, manual massage and chemical support with epinephrine were used. After this, the heart started to defibrillate. When a defibrillator was used, it started to beat in sinus rhythm. As the systolic pressure was low, an intra-aortic balloon was started while the patient was on bypass and dobutamine intravenous infusion. As the pressure and contractility started to build up, the patient was weaned from the bypass smoothly. Completion of the closure of the chest was performed conventionally, and the patient was taken with endotracheal intubation with chemical and mechanical support to the cardiac surgery intensive care unit.

After four hours, the patient was extubated successfully. All hemodynamic and biochemical parameters were within reference ranges for the first 24 hours. After that, the dobutamine dose reduced gradually, and the intra-aortic balloon support was weaned gradually over the first 24 hours. On the second postoperative day, the patient started to have a gradual decrease in his urine output despite intravenous replacement and oral intake. Chemical support with dobutamine was increased, and intra-aortic balloon reinstituted. An echocardiographic exam was performed, and it showed a clear pericardium and a normally functioning mechanical mitral valve but a failing LV with feeble contraction. The arterial pressure then started to go down gradually despite adding intravenous epinephrine to the support. Extracorporeal membrane oxygenation was initiated to help the failing heart. After 72 hours, the patient entered severe hypotension despite chemical and mechanical support. The patient was sedated and reintubated, and, as there was nothing surgical to correct, we kept him on high support in his third postoperative day. At the end of the third day, he developed resistant ventricular fibrillation, which did not respond to resuscitation through open massage.

## Discussion

Compared to a double-chambered RV, a DCLV is a rare anomaly with only very few reported cases in which the LV is partially divided into inlet and outlet portions [1,2,6]. It is a true division of the LV cavity as a result of the accessory septum, or muscle bundles [7]. It has been generally termed as DCLV with unknown etiology and is often associated with cardiomyopathy. Some relate the pathogenesis to hypoplasia of regional myocardial intratrabecular sinusoids, endocardial fibroelastosis, or LV non-compaction with cardiomyopathy [2,4,10]. It should be differentiated from a congenital LV diverticulum or aneurysm where synchronous contraction is considered the feature that serves to distinguish the DCLV from an aneurysm [10,11]. Distinguishing among these conditions is challenging but of great clinical importance, given the consequences of different treatment modalities and prognosis [4]. Usually, it is diagnosed during infancy or childhood, yet late presentation is possible and has been reported [1,2,4,11]. When detected in an asymptomatic adult and not associated with other cardiovascular abnormalities, the disorder is generally believed to be of little risk [4]. However, today data regarding an LV accessory chamber are sparse in the literature, and the prognosis is not well defined. A patient may develop severe heart failure requiring a heart transplant, life-threatening arrhythmias, and cardiac thromboembolism. The literature did not show what would be the best surgical solution for the late presentation of DCLV and heart failure when there is no access to a heart transplant. We thought, without access to a heart transplant, replacing the regurgitant valve with a mechanical valve and releasing the tethered LV might help to restore the LV contractility with the help of anti-failure medications. Because of the late presentation, it was not clear to us whether the mitral regurgitation is part of the pathology itself or secondary to the cardiomyopathy or both.

## Conclusions

A DCLV is an extremely rare cardiac anomaly with variable pathogenesis. It can present with mild symptoms that do not require special treatment, or it may present with severe cardiomyopathy and may be better to be treated surgically with a heart transplant rather than resection of the band and correcting the associated valve pathology.
